# Recurrence and satisfaction with sutured surgical treatment of an ingrown toenail

**DOI:** 10.1016/j.amsu.2020.06.029

**Published:** 2020-06-26

**Authors:** Mikołaj Dąbrowski, Anna Litowińska

**Affiliations:** a Department of Spine Orthopedics and Biomechanics, Poznan University of Medical Sciences, Poznan, Poland; b Anmedica-Healthy Foot Center, Poznan, Poland

**Keywords:** Ingrown toenail, Surgical technique, Preservation nail and matrix, Recurrence, Satisfaction, Esthetic results

## Abstract

**Background:**

In the present study, we investigated the satisfaction of patients following sutured surgical treatment of an ingrown toenail with nail preservation and without matricectomy.

**Materials and methods:**

This study was retrospective. In total, 37 consecutive patients underwent 54 ingrown toenail surgeries. The clinical outcomes, satisfaction of surgery, recurrence rates and the duration of symptoms were compared.

**Results:**

Recurrence with the technique was very low (one toenail). The mean overall satisfaction score on the surgical satisfaction questionnaire was 86.4 ± 10.4 and extended with a modified esthetic subscale (88 ± 10). The mean pain subscale score was the lowest at 77.1 ± 16.8, while the subscale returns to baseline scored 80.9 ± 16.4, the subscale global satisfaction scored 98.1 ± 7.2 and the subscale esthetics scored 92.1 ± 15.

**Conclusion:**

Our suturing technique was associated with low recurrence and high satisfaction rates. We showed that higher levels of satisfaction with the treatment were achieved in men, and the duration of symptoms was no longer than one year.

## Highlights

• This sutured surgery technique for ingrown toenails with the complete preservation of the nail and matrix led to high patient satisfaction with the therapeutic and esthetic results.
• This approach preserved nail function to improve both therapeutic and cosmetic results.

## 1 Introduction

Ingrown toenail (unguis incarnates, onychocryptosis) is a common condition in young adults [1,2]. Heifetz classification of ingrown toenails is divided into three stages: first stage is swelling of the nail grooves, second – acute infection and third is chronic infection with granulation tissue [3,4].

Risk factors for ingrown toenails include improper nail trimming, trauma, history of nail surgery, constrictive footwear, obesity, bony abnormalities, hyperhidrosis and diabetes [5]. This causes the nail to embed itself even further into the soft tissue, leading to a vicious cycle of recurrent swelling, pain and infection [6].

Many treatments have been proposed for ingrown toenails [6]. When non-surgical treatment is ineffective, surgery is required. Selective proximal matricectomy is a standard technique in first stage of disease. There are many different surgical techniques available for ingrown nails [7]. The most common surgical technique in hypertrophy nail fold is to remove part of the ingrown nail and nail fold [8]. During wedge cutting, partial matricectomy is performed, which often involves the destruction of part of the nailbed [1,6,9]. Segmental excision, complete nail removal and partial nail removal have been combined with matricectomy (surgical or chemical) to reduce the risk of recurrence. This surgical technique is aimed at destroying the nail matrix in addition to removing the troublesome part of the nail. The use of chemical ablation reduces the recurrence rate regardless of the type of surgery. Recurrence rates were 20% with wedge resection alone, while with chemical ablation this was reduced to 11%. Wedge excision showed a similar frequency of relapses as complete nail removal, but was more effective than foldoplasty [10]. The effects of wedge operations or radical treatments do not meet the esthetic requirements of young adults.

Surgery techniques in cases hypertrophy nail fold with the preservation of the nail and matrix are not often used. We have found that the nail plate itself is a good point for skin plasty. We were interested in whether a satisfactory result could be obtained without removal or partial resection of the nail plate, i.e. with only soft tissue resection and skin plasty. Our goal was to evaluate the results of ingrown nail corrections in patients who underwent our surgical approach in terms of healing time, pain relief, relapse and scarring.

## 2 Materials and Methods

### 2.1 Study design

The study was a retrospective study of children, adolescents or adults who underwent our procedure with preservation of the nail and matrix for one or more ingrown toenails. The study was reported in line with the PROCESS criteria [11]. The study was registered under number ChiCTR2000029649 [12].

### 2.2 Participants

Participants in the study were included if they were children, adolescents or adults who decided to undergo our procedure with preservation of the nail and matrix for one or more ingrown toenails. Individuals were excluded if they could not understand Polish or had significant medical comorbidities. Participants represented a convenience sample identified through the outpatient surgical clinic as private practice at Anmedica-Healthy Foot Center (Poznan, Poland).

Based on clinical volume, we anticipated being able to recruit approximately 40 participants over a two-year period. All participants underwent consultation with one podiatric nurse. Written consent was obtained from the patient; for juvenile participants, written consent was obtained from the parent or legal guardian as well.

The baseline assessment included questions regarding the duration of symptoms, risk factors and previous treatment. The surgeon also classified the severity of disease using the Heifetz classification system: stage 1 – slight erythema and swelling of the nail grooves in the nail bed, stage 2 – presence of acute infection and suppuration, stage 3 – chronic infection, the formation of granulation tissue surrounding the nail groove and hypertrophy of the surrounding tissues [3]. Two patients had hypertension. Other patients did not have comorbidities.

### 2.3 Intervention

All participants underwent the procedure with preservation of the nail and matrix for one or more ingrown toenails as an outpatient day procedure approximately one to two months after their initial consultation. The procedure was performed in the operating room under local anesthetic. All procedures were performed by one orthopedic surgeon with prior experience with the procedure with the preservation of the nail and matrix. The first author has 8 years of experience in spine surgery (Wiktor Dega Hospital in Poznan, Poland) and in orthopedic surgery and 5 years of experience in nail surgery.

### 2.4 Surgical technique

#### 2.4.1 Preparation for the procedure

In order to qualify for surgery, each patient received a health questionnaire, which was sent to the Center, along with photographs. The patients received periprocedural recommendations: an inventory of dressing materials, a method for preparing the toe for surgery, as well as postoperative procedures, including instructions for dressing changes. They also received recommendations that, for three consecutive days before the procedure, the toe should be soaked for 10 min in a prepared solution of potassium permanganate, then dried and lightly bandaged. On the day of the surgery, the patient reported with the toe secured by a bandage.

#### 2.4.2 Anesthesia

First, the involved toe was prepared in a sterile fashion, and ring nerve block anesthesia was applied using 2% lidocaine without epinephrine. After the anesthesia procedure, an elastic tourniquet was placed at the base of the toe to maintain a clear and bloodless surgical field.

#### 2.4.3 Surgical procedure

**Fig. 1:**
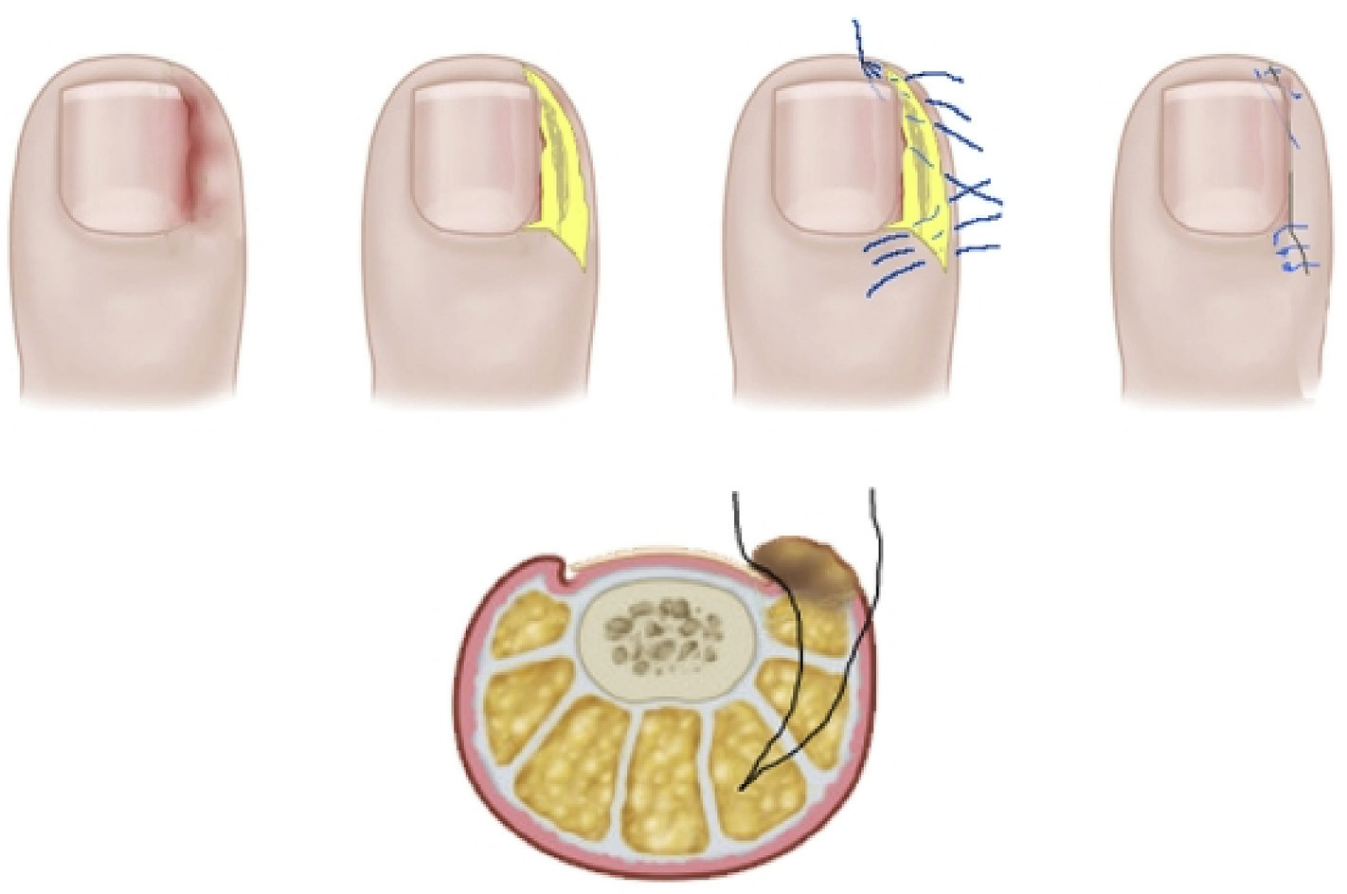
Surgical technique for the treatment of an ingrown toenail with preservation the nail and matrix.

The operational approach was a modification of the Noel procedure [13]. The area to be excised was outlined with a skin marker. A wedge-shaped ellipse of soft tissue, including the fibrotic and granulation tissue, was removed on both sides of the nail (Fig. 1). The incision lines were adjacent to the lateral borders of the nail plate and continued distally for approximately 4–6 mm. While proximal to the cutting is performed parallel to the rear of the nail at a distance of approximately 1/4 - 1/5 of the width of the nail. The lateral cut was semi-elliptical [7], and its extent depended on the size of inflammatory lesion. Incisions were deep enough to remove a large volume of soft tissue (Fig. 1).

**Fig. 2:**
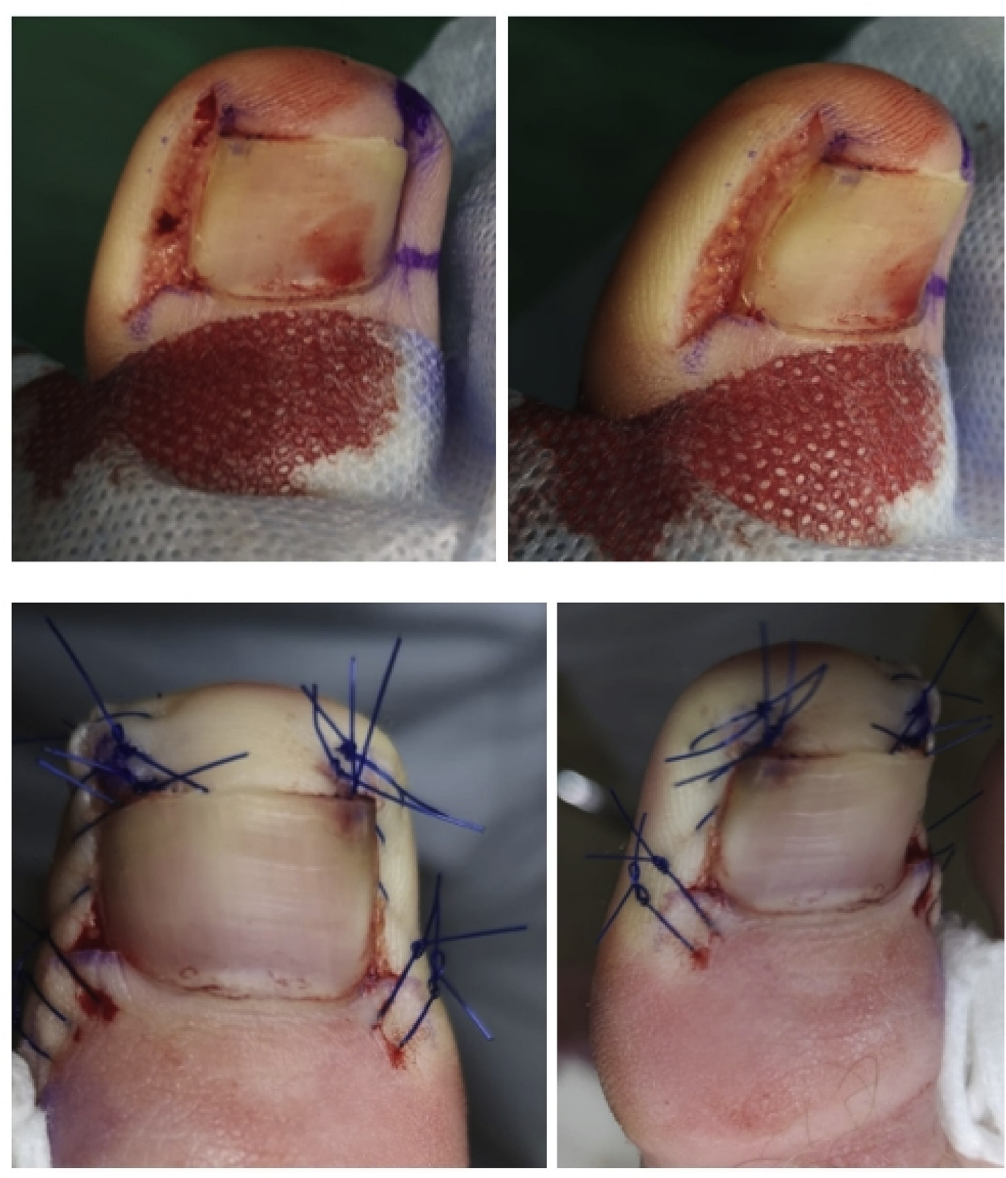
Surgical technique the during operation.

The defect was closed with simple interrupted 4-0 polypropylene sutures. We introduced a subungual suture in the middle of the lateral margin of the wound, conducting it in the subcutaneous tissues and ending the suture distal from the hyponychium (Fig. 2).

Further surgical sutures were placed in the distal direction. This surgical suture ensured that the lateral edge of the wound was pulled under the edge of the nail [13,14]. Proximal surgical sutures approached the edges of the wound. No nail avulsion was performed. Care was taken to avoid injury to the nail bed and nail matrix.

A sterile dressing was placed; this was removed by the patient after 48 h. The patient changed subsequent dressings every 24 h. The average duration of surgery was approximately 25–40 min, including the waiting time for anesthesia.

#### 2.4.4 After surgery

Patients were discharged home about 30 min after surgery. They received an information card and recommendations for handling the wound. If the surgery involved two toes, the patient was given 50 mg ketoprofen orally after the first toe operation.

In the case of blood congestion of the dressing, a further layer of dressing was applied. For two days after the operation, patients were instructed not to soak the surgical area in water. Patients could wash, provided the involved foot remained outside the bath or shower. They were also told that they should not overload the forefoot for the first three days; however, they could and should walk. Until the day the sutures were removed (about 12–14 days), wearing of full or tight shoes was not recommended. After about 48 h, the first dressing change occurred, with subsequent changes every 24 h. If there was no excessive bleeding or exudate, the dressing was gradually decreased. For 3–5 days after removal of the stitches, the patient continued to use dressings. On subsequent days, the use of an anti-scarring preparation was mandatory. To facilitate complete healing, using a swimming pool, sauna, or solarium, participating in sports and wearing tight shoes were not allowed. At about 48 h, when the patient changed the dressing for the first time, control photographs of the surgical area were taken and sent to the Center by email.

### 2.5 Outcomes

Participants were assessed at a mean time point of 11 months postoperatively. The primary outcome for the study was recurrence. This was defined as: “Has the problem you had surgically corrected reappeared at the same location?”, which was the absence of a definitive cure. Two questions were asked with possible answers being either “yes” or ”no”: “Has the problem you had surgically corrected recurrence at the same location?” and “Was further medical or surgical treatment required in the area where the surgery was performed?”.

Secondary outcomes included complications, pain, and patient satisfaction. Complications were assessed by asking to participants to record any clinical sequelae related to surgery that prompted a phone call to the surgeon's office, additional clinic appointment or visit to the emergency department. Examples included excessive pain, infection, bleeding and wound care issues. In addition, two questions were asked with possible answers being either “yes” or ”no”: “Have you experienced complications?” and “Is there any abnormal innervation of the tip of the toe?”.

To assess the surgical procedure and obtain clinical information, we used a questionnaire. Participants were asked to complete the surgical satisfaction questionnaire (SSQ) [9,15]. This instrument consists of eight items related the patient personal satisfaction with the procedure and outcome (e.g., “Would you recommend this surgery to someone else?”). The standardized questionnaire was mailed to all patients and included a patient self-assessment with responses recorded on a five-point scale. The questions were scored with a value of 4 for “very satisfied” to a value of 0 for “very unsatisfied”. The mean average of the scores was multiplied by 25 (the questionnaire was considered incomplete if more than two items were not answered), yielding a potential range of scores from 0 to 100. The higher the score, the greater the degree of surgical satisfaction. Items 1 and 2 were used to calculate the Pain subscale; items 3, 4 and 5 were used for the Return to baseline subscale; and items 6, 7 and 8 were used for the Global satisfaction subscale. We added three questions about the appearance of the toe after surgery, from which we calculated the esthetic subscale: “How satisfied are you with the esthetic result after surgery?”, “How satisfied are you with the current appearance of the nail folds?” and “How satisfied are you with the appearance of the postoperative scar?”.

Pain was assessed on an 11-point numeric rating scale (NRS) twice. The first measurement was taken before surgery. Another measurement was obtained while collecting data on the satisfaction with the procedure.

### 2.6 Statistical analysis

Statistica 13.0 software (StatCorp., College Station, TX, USA) and Microsoft Excel 2016 were used in the analysis. Descriptive values of variables were expressed as means ± standard deviation or medians (minimum-maximum). Statistical significance was set at p < 0.05. Dichotomous data were presented as counts and frequencies and continuous data as means and standard deviation (SD). To compare the impact of various factors, a *t*-test was performed.

## 3 Results

### 3.1 Baseline characteristics

We retrospectively reviewed the charts of 37 consecutive patients who underwent 54 ingrown toenail surgeries between 2017 and 2019. The average follow-up time was 11.6 months. Age ranged from 12 to 71 years with a mean of 25.2 (SD = 12.7) years. The half of participants were between 17 and 25 years of age. Men predominated (67.6%); there were 25 males and 12 females. At the time of their initial consultation, participants reported having symptoms for under one year in 16 patients (29.6%), two to three years in 24 patients (44.4%) and more than three years in 14 patients (25.9%).

Twenty-three participants (62.2%) indicated that their risk factor was improperly trimmed nails and poorly fitting shoes was implicated in 15 patients (40.5%).

The participants had significant disease based on the Heifetz classification system: 5 patients (9.2%) were stage I, 20 patients (37.1%) were stage II and 29 patients (53.7%) were stage III (Table 1).

**Table 1:** Characteristics of the study group.

Parameter		Results
Age [years] (n = 37)	Mean ± SD	25.2 ± 12.7
	Median (min-max)	22 (12-71)
Sex (n = 37)	Female	12 (32.4%)
	Male	25 (67.6%)
Observation time [months] (n = 37)	Mean ± SD	11.6 ± 6.7
	Median (min-max)	9.2 (2.2-28.1)
Toes operated (n = 37)	Unilaterally	
	Right	11
	Left	10
	Both sides	16
Side of toe involvement (n = 54)	Medial	3
	Lateral	6
	Both	45
Duration of symptoms [years] (n = 54)	<1	16 (29.6%)
	2–3	24 (44.4%)
	>3	14 (25.9%)
Risk factors (n = 54)	Improperly trimmed nails	23 (62.2%)
	Poorly fitting shoes	15 (40.5%)
	Trauma	6 (16.2%)
	Hyperhidrosis	5 (13.5%)
	Overweight/obesity	5 (13.5%)
	Anatomic abnormalities	2 (5.4%)
Previous treatment (n = 54)	Surgery – wedge segmental excision	16 (29.6%)
	Nail claws	10 (18.5%)
	Surgery – removal of the nail	9 (16.7%)
	Antibiotics	9 (16.7%)
	Arcada's cube	4 (7.4%)
	Dermatologist – antifungal treatment	2 (3.7%)
	Nail plate reconstruction	1 (1.9%)
	Surgery – ingrown nail correction	1 (1.9%)
	Not treated	16 (29.6%)
Heifetz stage (n = 54)	Stage 1	5 (9.2%)
	Stage 2	20 (37.1%)
	Stage 3	29 (53.7%)

SD, standard deviation.

Before the surgical intervention, detailed medical histories were obtained from all patients in this case series, and physical examinations were performed. No exclusion criteria were set, and a consecutive cohort of patients was used.

### 3.2 Recurrence

We further sought to ensure appropriate responses by asking patients if the problem they had surgically corrected reappeared at the same location. In our cohort, only one patient reported that the problem had recurred, but surgical treatment was not required in the area (Table 2).

**Table 2:** Numbers and percentage shares of responses to questions regarding the operative treatment of ingrown toenail in patients (n = 54).

Has the problem you had surgically corrected reappeared at the same location?	Yes	1
	No	53
Have you experienced complications?	Yes	4
	No	50
Is there any abnormal innervation of the tip of the toe?	Yes	2
	No	52

### 3.3 Complications

Five participants experienced one or more minor complications after surgery. Complications occurred during one toe operation; it was a problem with a postoperative hematoma and prolonged healing. In four cases, there was necrosis of the skin, which subsequently healed. Abnormal innervation of the tip of the toe occurred in two patients.

### 3.4 Patient satisfaction

All participants returned to the clinic six months postoperatively and completed the Surgical Satisfaction Questionnaire (Table 2). 96% indicated that they would have the surgery again if they had to do it all over again and would recommend the procedure to someone else. The lowest ratings were seen in pain was controlled after returned home after surgery, with 37% of participants reporting neutral or unsatisfied responses (Table A1).

The mean overall satisfaction score from the SSQ procedure was 86.4 ± 10.4 and extended with a modified esthetic subscale (88 ± 10). The mean Pain subscale score was the lowest at 77.1 ± 16.8, while the subscale Return to baseline scored 80.9 ± 16.4, the subscale Global satisfaction scored 98.1 ± 7.2 and the subscale Esthetics scored 92.1 ± 15 (Table 3).

**Table 3:** Comparison of surgical satisfaction questionnaire score, as well as pre- and postoperative pain according to the NRS according to gender.

Instrument	Total (N = 54)	Women (n = 12)	Men (n = 25)	p
SSQ total score	86.4 ± 10.4	**80.5 ± 11.6**	**89.1 ± 8.7**	**<0.01**
SSQ total score with esthetic subscale	88 ± 10	**82.9 ± 10.9**	**90.3 ± 8.8**	**0.01**
SSQ subscales				
Pain	77.1 ± 16.8	70.6 ± 20.2	80.1 ± 14.3	0.05
Return to baseline	80.9 ± 16.4	**71.1 ± 15.6**	**85.4 ± 14.9**	**<0.01**
Global satisfaction	98.1 ± 7.2	96.6 ± 12.2	98.9 ± 2.9	0.28
Esthetic	92.1 ± 15	89.2 ± 17.6	93.5 ± 13.6	0.34
Preoperative pain (NRS)	7.1 ± 2.7	**8.4 ± 2**	**6.6 ± 2.8**	**0.02**
Postoperative pain (NRS)	0.4 ± 1.1	0.2 ± 0.7	0.5 ± 1.2	0.34

**Fig. 3:**
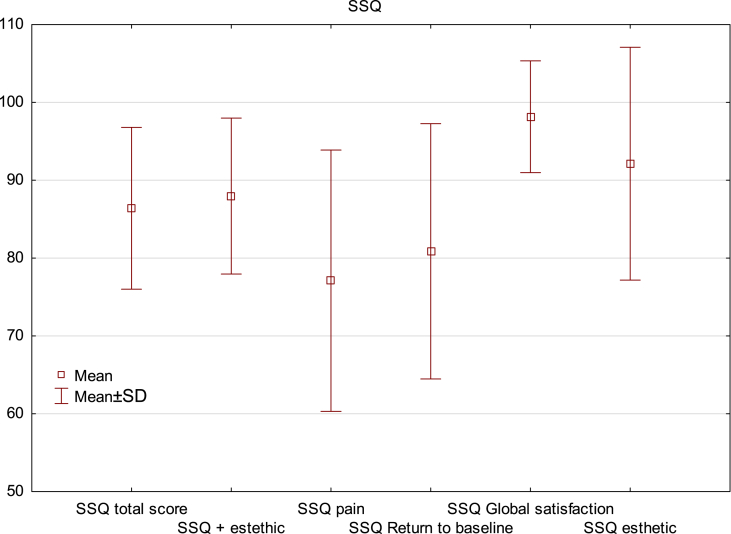
Postoperative responses of patients who underwent surgical treatment for an ingrown toenail. Graph of the mean values and standard deviations of the answers to questions regarding satisfaction with the surgery.

Men achieved a significantly higher total score in the SSQ than women (89.1 ± 8.7 vs. 80.5 ± 11.6, p < 0.01). Women were significantly less satisfied on the Return to baseline subscale (71.1 ± 15.6 vs. 85.4 ± 14.9, p < 0.01). There were no differences between sex in the Global satisfaction (96.6 ± 12.2 vs. 98.9 ± 2.9, p = 0.28) or esthetic (89.2 ± 17.6 vs. 93.5 ± 13.6, p = 0.34) subscales. Interestingly, women were less satisfied with pain control (70.6 ± 20.2 vs. 80.1 ± 14.3, p = 0.05), and preoperative pain intensity (NRS) was significantly higher in women than in men (8.4 ± 26.vs. 6.6 ± 2.8, p = 0.02) (Table 3, Fig. 3).

**Fig. 4:**
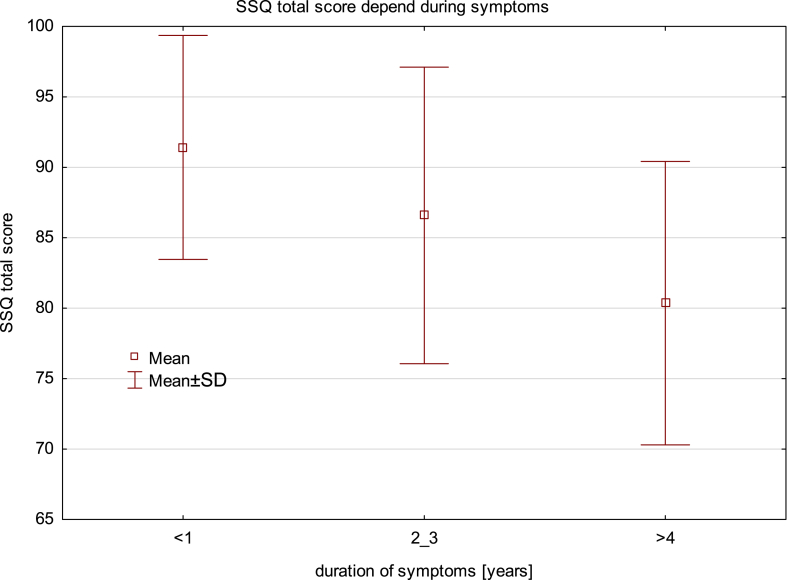
Graph of means and standard deviation of the surgical satisfaction questionnaire total score depending on the duration of symptoms.

A significantly higher total score of satisfaction according to the SSQ was achieved in patients with a shorter duration of symptoms (91.4 ± 7.9 vs. 84.3 ± 10.7, p = 0.02). The duration of symptoms over a year gave significantly worse satisfaction with return to baseline (89.1 ± 11.3 vs. 77.4 ± 17.1, p = 0.02). There were no differences based on the duration of symptoms regarding final global satisfaction on the surgery subscale (99.0 ± 2.8 vs. 97.8 ± 8.4, p = 0.6) or the esthetic result (94.8 ± 9.6 vs. 91 ± 16.7, p = 0.4) (Table 4, Fig. 4).

**Table 4:** Comparison of surgical satisfaction questionnaire score, pre- and postoperative pain according to the NRS regarding the duration of symptoms.

Instrument	Duration of symptoms		p
	<1 year	≥1 year	
SSQ total score	**91.4 ± 7.9**	**84.3 ± 10.7**	**0.02**
SSQ total score with esthetic subscale	**92.3 ± 7.3**	**86.1 ± 10.5**	**0.04**
SSQ subscales			
Pain	83.6 ± 15.6	74.3 ± 16.7	0.06
Return to baseline	**89.1 ± 11.3**	**77.4 ± 17.1**	**0.02**
Global satisfaction	99.0 ± 2.8	97.8 ± 8.4	0.6
Esthetic	94.8 ± 9.6	91.0 ± 16.7	0.4
Preoperative pain (NRS)	6,6 ± 3	7,3 ± 2,6	0,38
Postoperative pain (NRS)	0,1 ± 0,3	0,5 ± 1,3	0,25

## 4 Discussion

### 4.1 Surgical technique

Our results suggest that this procedure with preservation of the nail and matrix is associated with a low recurrence rate among adolescents and adults with ingrown toenails. Five participants experienced complications of surgery. These included bleeding and excessive pain that prompted a phone call/email to the clinical office or an extra clinical appointment. None of these complications required re-surgery.

Similar findings (mean age of 21.3 years) were presented by Abdel-Maksoud, who used the technique of lateral nail-fold excision without matricectomy with closure by secondary intention in the treatment of ingrown toenails. The study was a prospective observational study carried out on 83 patients. The recurrence rate was 3.6%, postoperative erythema and swelling was 3.6%, inflammatory exudate was 4.8% and loss of sensation around the area of surgery was 2.4%. 94% of patients were highly satisfied with the procedure [16].

Chapeskie et al. evaluated a procedure with excision of excessive nail-fold granulation tissue with preservation of the nail and its matrix in 124 mixed adult-pediatric patients. The wound was then allowed to close via secondary intention. They had excellent cosmetic results, no recurrences after 12 months and 94.3% patient satisfaction [17].

The release of the nail by a removing a sufficiently large volume of the surrounding nail plate results in a reduction in inflammation and promotes healing. It is not necessary to remove the nail bed and/or perform a matricectomy. Treatment of an ingrown nail by decompression of the nail without removal of the matrix has been found to be effective in recent studies [13,18].

Wedge excision of the side wall of the nail on the affected side may be sufficient for decompression of the nail, especially in advanced cases of ingrown nails. Removal of the appropriate volume of soft tissue in the nail shaft is the essence of the procedure and should be tailored to the needs of the patient, especially with an advanced degree of ingrown nail. Removing more soft tissue reduces the recurrence rate and improves the cosmetic appearance of the toe [13].

Many recurrences may be due to insufficient removal of hypertrophic tissue surrounding the lateral parts of the nail. Partial removal of the placenta and nail matrix to improve this condition is not always a necessary solution. The effect of this treatment leads to a deformed toe with a narrow dystrophic nail. Even with a very narrow nail, there may be pain and, in order to achieve full improvement, sometimes it is necessary to completely remove the nail [13].

This method is using a procedure with preservation nail and matrix and closure by first intention. A similar knot technique was also used by Ince et al. who compared their technique with the Winograd method. The knot technique is a simple technique for treating ingrown nails with fewer complications and shorter surgery times. It consists of the removal of a soft tissue wedge without affecting the nail itself without the need for surgery on the nail bed. Three main factors have been shown to influence the success of this procedure: wedge excision combined with primary suturing, sufficient granulation tissue excision and sutures in the proximal part of the ingrown part of the nail [[19], [20], [21], [22]]. Recurrence rates are low, but secondary wound healing time may be delayed compared to other techniques. Delayed wound healing was also observed in another study in which soft tissue defects were reduced by primary suturing [20,23].

This surgical technique involved a surgical suture for the nail and paid attention to surgically removing or ablating lateral wall hypertrophy and granulation tissue. It had a high cure rate, short postoperative pain duration and low risk of postoperative infection, as well as very good esthetic results [24].

We noted the seam, regardless of whether it was applied to the nail or in a similar technique to Noel, i.e. with the skin fold under the nail. This technique reduced recurrences, had better operative outcomes and reduced recurrences and was associated with greater satisfaction [22].

Surgical principles specify that the sutures should not be used during tissue inflammation. Our experience in this respect partly deviates from these principles. This may be controversial, but in our opinion, the sutures provide the opportunity for primary union in most cases, and we put the sutures on each patient. Indeed, in patients with advanced tissue hyperplasia and chronic inflammation can have swollen scar tissue involving the nail shaft. Among these patients, a more common complication of healing is loosening or releasing sutures and secondary wound healing. However, in the first phase of healing, the surgical sutures were rarely loosened. Some sutures remained, which additionally led to less susceptibility to infection as in primary healing. In addition, the use of sutures, despite secondary healing, led to a more predictable nail shape after treatment.

**Fig. 5:**
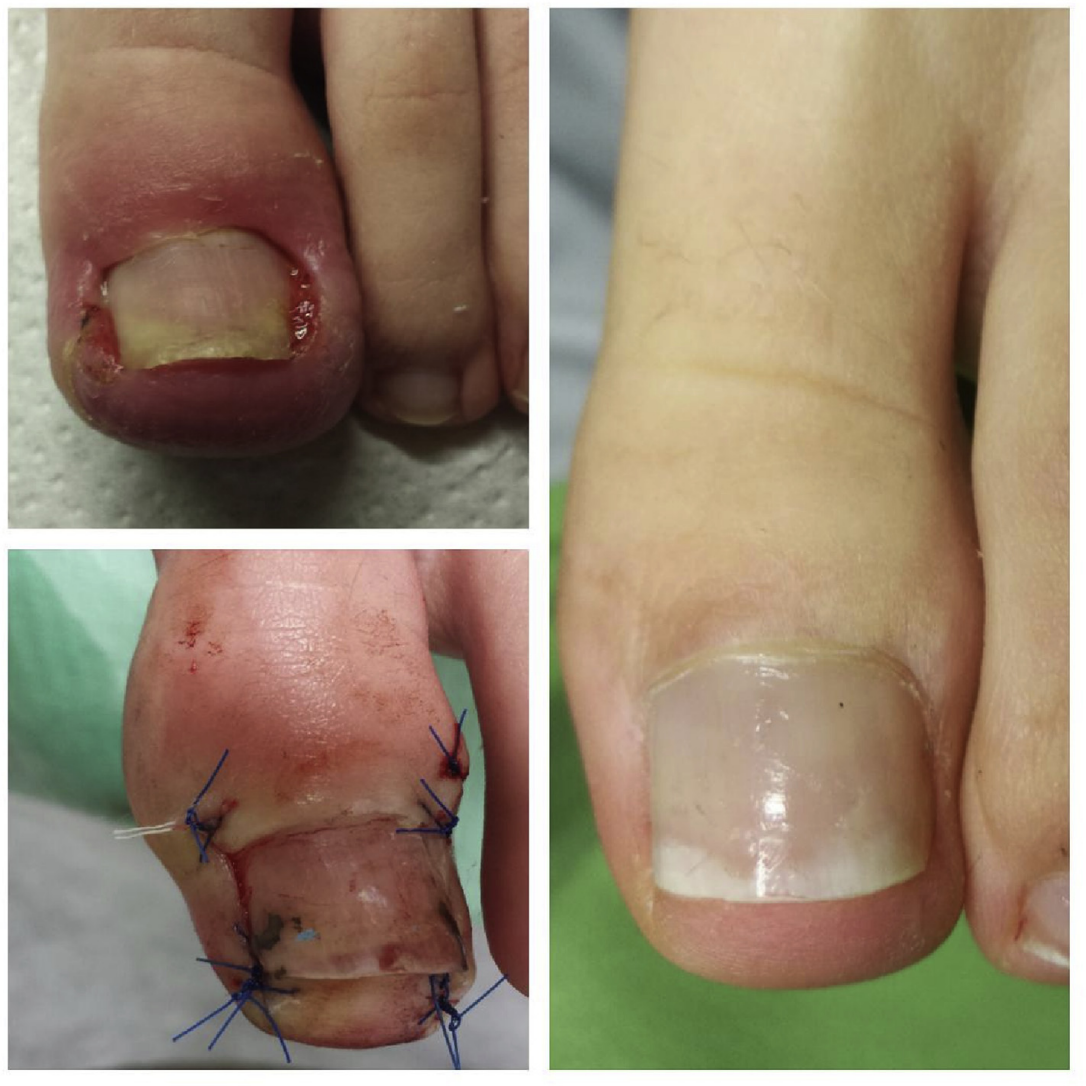
Clinical outcomes of treatment before and after surgery.

Another aspect of applying surgical sutures to the nail may be the development of a nail bed disorder; this is why we did not use nail sewing in our procedure, although is used in many currently applied methods. In the first phase after treatment, we obtained the effect of slimming the toe. However, based our experience, this condition is transient, and the cosmetic effect after two to three months is satisfactory to patients (Fig. 5). Moreover, a basic analgesic effect was achieved. Complications of this technique for ingrown nail surgery were low and depended to a large extent on the stage of the disease. The complication rate was comparable to the corresponding saving techniques described by other authors [13,20].

Loss of sensation at the surgical site was observed in two patients. A similar percentage was reported by other authors [25]. Postoperative therapy involves controlling the growth of the nail plate and the condition of the surgical site. Every one to two weeks, the podologist cleans the nail and surgical site, checking whether the patient is following the care instructions. Ince et al. emphasized the importance of the postoperative period. Patients should follow the standard anti-infection protocol, avoid tight shoes and not cut their nails for two months [20].

### 4.2 Patient satisfaction

We chose the SSQ to evaluate the satisfaction with the procedure because it was previously used to evaluate after ingrown nail treatments and seemed objective and easy to perform [9,15]. A high level of satisfaction with the procedure was demonstrated. Global satisfaction and esthetics were very high at the expense of less satisfaction with pain control and return to activity. Following surgery of the foot and toes, this is a frequent problem due to the high degree of innervation and prolonged healing. We observed that preoperative pain was significantly more severe in the Heifetz group III patients. However, the stage of the disease did not have a significant impact on the patient's satisfaction with the procedure.

In previous publications, no evidence of gender-based differences in patient satisfaction with ingrown nail surgery has been found. However, in the present study, men had a significantly higher level of satisfaction with the procedure compared to women. The difference was in the SSQ subscale on returning to daily activities, in which men definitely described a greater degree of satisfaction. This may also be due to the fact that, among men, there was a higher percentage of patients with stage III (approximately 60%) compared to women (40%). In addition, this study showed a significant difference in patient satisfaction after surgery depending on the duration of symptoms. The shorter the symptoms (<1 year), the greater the satisfaction with the procedure. This may mean that we should not extend various conservative therapies and podologists, in the event of ineffectiveness, should quickly decide to send the patient to the podiatric surgery center.

The Polish population is dominated by patients with a more advanced (II and III) stage of the disease. Therefore, cases with hypertrophy of the nail fold dominate. That is why we rarely use selective proximal matricectomy. Furthermore, patients seek help when it comes to very advanced scarring and filtration wounds. Thirdly lingers still use the old surgical methods including cutting wedge that give poor surgical effects. That is why there is a delay in non-surgical treatment in our country. The situation is slowly increasing number of podiatrists. Conservative treatment, e.g. with clamps, is at an ever higher level, but more time is needed to increase public awareness in this respect.

Further research is aimed at increasing the group of patients with repeated assessment of treatment satisfaction and at the same time extending the observation time.

### 4.3 Limitations

There are several limitations to this study. The sample size was small and, due to low statistical power, the results may not show all possible differences in statistical significance. In addition, the used treatment satisfaction questionnaires present a subjective assessment of patients. There is no objective clinical assessment of the condition of the nail after surgery.

## 5 Conclusions

In conclusion, during the follow-up period, one recurrence was reported. We suggest that ingrown toenails with hypertrophy nail fold can be surgically treated without matricectomy with good cosmetic results. The advantage of the presented technique is the complete preservation of periungual area anatomy with simultaneous high patient satisfaction with the therapeutic and esthetic results. Our results are similar to those published by other researchers using analogous surgical techniques. We noted better results in patients with a shorter duration of symptoms, which supports the faster qualification of patients for surgery. In addition, more satisfactory results were found in men in terms of pain and return to baseline.

## Ethical approval

The work was approved by the appropriate ethical committees related to the institution(s) in which it was performed. The patients provided informed consent to the work.

## Sources of funding

The funding source had no involvement.

## Author contribution

Conceptualization – MD. Data curation – MD. Formal analysis – MD. Funding acquisition – AL. Investigation – MD. Methodology – MD, AL. Project administration – MD, AL. Supervision – AL. Validation – AL. Roles/Writing of original draft – MD. Writing review & editing – MD, AL.

## Registration of research studies

Name of the registry: Chinese Clinical Trial Registry.
Unique Identifying number or registration ID: ChiCTR2000029649
Hyperlink to your specific registration (must be publicly accessible and will be checked): http://www.chictr.org.cn/showprojen.aspx?proj=47820

## Guarantor

Mikolaj Dabrowski

## Consent

Written informed consent was obtained from the patient for publication of this case report and accompanying images. A copy of the written consent is available for review by the Editor-in-Chief of this journal on request.

## Provenance and peer review

Not commissioned, externally peer reviewed.

## Declaration of competing interest

The authors have no competing interests to declare.

## Figures and Tables

**Table A1:** Participant ratings on the surgical satisfaction questionnaire after surgery.

Item	Response	Participant rating
How satisfied are you with how your pain was controlled in the hospital after surgery?	Satisfied Neutral/unsatisfied	89% 11%
How satisfied are you with how your pain was controlled when you returned home after surgery?	Satisfied Neutral/unsatisfied	63% 37%
How satisfied are you with the amount of time it took for you to return to your daily activities, for example housework or social activities outside the home?	Satisfied Neutral/unsatisfied	87% 13%
How satisfied are you with the amount of time it took for you to return to work?	Satisfied Neutral/unsatisfied	85% 15%
How satisfied are you with the amount of time it took for you to return to your normal exercise routine?	Satisfied Neutral/unsatisfied	87% 13%
How satisfied are you with the results of your surgery?	Satisfied Neutral/unsatisfied	98% 2%
Looking back, if you “had to do it all over again” would you have the surgery again?	Yes Maybe Unsure/do not think so/no	96% 2% 2%
Would you recommend this surgery to someone else?	Yes Maybe Unsure/do not think so/no	96% 2% 0
How satisfied are you with the esthetic results after surgery?	Satisfied Neutral/unsatisfied	96% 4%
How satisfied are you the current appearance of the nail folds?	Satisfied Neutral/unsatisfied	98% 2%
How satisfied are you the appearance of the postoperative scar?	Satisfied Neutral/unsatisfied	94% 6%

**Table A2:** Comparison of surgical satisfaction questionnaire responses according to Heifetz stage

Instrument	I and II° (n = 25)	III° (n = 29)	p
SSQ total score	86.5 ± 12.5	86.3 ± 8.4	0.95
SSQ total score with esthetic subscale	88.3 ± 10.5	87.7 ± 9.8	0.84
SSQ subscales			
Pain	80 ± 16.5	74.6 ± 16.9	0.24
Return to baseline	80 ± 18.8	81.6 ± 14.3	0.72
Global satisfaction	97.3 ± 10.1	98.9 ± 2.9	0.44
Esthetics	93 ± 9.8	91.4 ± 18.4	0.70
Preoperative pain in NRS	**5.8 ± 2.9**	**8.2 ± 2**	**<0.01**
Postoperative pain in NRS	0.2 ± 0.6	0.6 ± 1.4	0.24
